# Neuropilin-1 and the Positions of Glomeruli in the Mouse Olfactory Bulb

**DOI:** 10.1523/ENEURO.0123-16.2016

**Published:** 2016-11-01

**Authors:** Bolek Zapiec, Olaf Christian Bressel, Mona Khan, Andreas Walz, Peter Mombaerts

**Affiliations:** Max Planck Research Unit for Neurogenetics, 60438 Frankfurt, Germany

**Keywords:** axon guidance, bulb, cAMP, neuropilin, odorant, olfactory

## Abstract

It is known since 1996 that mouse odorant receptors (ORs) are involved in determining the positions of the sites of coalescence of axons of olfactory sensory neurons (OSNs)—the thousands of glomeruli in the olfactory bulb. But the molecular and cellular mechanisms of OR-mediated axonal coalescence into glomeruli remain unclear. A model was proposed in 2006–2009 whereby OR-derived cAMP signals, rather than direct action of OR molecules, determine the target destinations (glomeruli) of OSNs in the bulb. This model hypothesizes that OR-derived cAMP signals determine the expression levels of neuropilin 1 (Nrp1) in OSN axon termini; that levels of Nrp1 in glomeruli form a gradient from anterior-low to posterior-high throughout the bulb; and that these Nrp1 levels mechanistically determine anterior-posterior patterning of glomeruli. Here, we describe the first independent evaluation of the Nrp1 model since it was formulated a decade ago. We tested the model for the well-characterized mouse OR M71 using our gene-targeted mouse strains, which are publicly available. In contradiction to the model, we observed a variety of configurations for the M71 glomeruli in the conditional *Nrp1* knockout. We then reassessed the model for the original OR transgene with which the model was developed, using the same publicly available mouse strains. We discovered that glomerular positions do not undergo the simple anterior shift that has been reported in the conditional *Nrp1* knockout for this OR transgene. Taken together, our findings do not support the Nrp1 model for the anterior-posterior patterning of glomerular positions in the olfactory bulb.

## Significance Statement

In the mouse, each olfactory sensory neuron expresses one of ∼1100 odorant receptor genes. The odorant receptor determines to which odorants the neuron responds physiologically and in which glomerulus of the olfactory bulb its axon terminates. A model was proposed 10 years ago whereby intracellular signals derived from the odorant receptor determine the level of neuropilin 1, which in turn determines the position of the glomerulus along the anterior-posterior axis of the bulb. We provide the first test of this model, for a well-characterized odorant receptor and separately with the original mouse strains that led to the formulation of the model. Our results do not support the neuropilin 1 model of anterior-posterior patterning of glomerular positions in the olfactory bulb.

## Introduction

In the mouse, the 1100 odorant receptor (OR) genes (Buck and Axel, 1991) determine to which odorants an olfactory sensory neuron (OSN) responds (Bozza et al., 2002) and in which of the 3600 glomeruli of the olfactory bulb (Richard et al., 2010) the axon of an OSN terminates (Mombaerts, 2006). A gene-targeted replacement of the OR coding region provided the first evidence that ORs are mechanistically involved in determining the positions of glomeruli (Mombaerts et al., 1996). A set of experiments with a transgenic mouse MOR23 promoter defined by Vassalli et al. (2002), but expressing a rat OR (I7) instead of mouse MOR23, led to a model whereby OR-derived cAMP signals, rather than direct action of OR molecules, determine the target destinations (glomeruli) of OSNs (Imai et al., 2006, 2009; Luo, 2015). This model proposes that OR-derived cAMP signals regulate the transcription of genes encoding axon guidance molecules, which then guide positioning of glomeruli along the anterior-posterior axis of the bulb. OSNs expressing rat OR I7 from a transgenic mouse MOR23 promoter form, in the medial aspect of the bulb, a novel, artificial glomerulus, which appears to be homogeneous in that it is not coinnervated by OSNs expressing an endogenous mouse OR (Imai et al., 2006, 2009). Immunostaining in bulb sections for the axon guidance molecule neuropilin 1 (Nrp1) in mice at postnatal day 14 (PD14) revealed, in the medial aspect of the bulb, a gradient of Nrp1 expression in a row of 20 glomeruli that encompass the novel, artificial glomerulus, with low glomerular Nrp1 expression at the anterior end and high expression at the posterior end (Imai et al., 2006). By crossing a conditional *Nrp1* knockout (Gu et al., 2003) with a transgene-expressing rat OR I7 from a mouse MOR23 promoter along with Cre recombinase, an anterior glomerular shift was reported at PD14; conversely, when Nrp1 was overexpressed in these OSNs from a transgene, a posterior glomerular shift was reported (Imai et al., 2009). Agonist-independent G protein–coupled receptor activity was later proposed to regulate anterior-posterior targeting of axons of OSNs via Nrp1 (Nakashima et al., 2013).

Curiously, when formulating the Nrp1 model, data were reported only for the projection sites (glomeruli) in the medial aspect of the bulb (Imai et al., 2006, 2009). It is well established that glomeruli for a given OR, with a few exceptions (Strotmann et al., 2000), are found in both medial and lateral aspects of the bulb, including the MOR23 glomeruli (Vassalli et al., 2002; Zapiec and Mombaerts, 2015). Thus, it is not known what impact the conditional *Nrp1* knockout has on the lateral glomerulus in mice that express rat OR I7 from a transgenic mouse MOR23 promoter.

Here, we describe the first independent evaluation of the Nrp1 model since it was formulated a decade ago. We tested the model with a genetic strategy based on the well-characterized mouse OR, M71. The M71 glomeruli reside posteriorly and are Nrp1^+^ (Dibattista and Reisert, 2016). Our experimental design makes use of our publicly available mouse strains that carry gene-targeted mutations. We avoided the use of small transgenes, because these are notorious for their line-to-line variability (Vassalli et al., 2002; Vassalli et al., 2011). Moreover, these gene-targeted strains enable us to study glomeruli that are formed by the coalescence of axons of OSNs that express a mouse OR from its endogenous locus. We also created a mosaic situation whereby Nrp1^+^ M71^+^ OSNs coexist in the same mouse with Nrp1^–^ M71^+^ axons, capitalizing on the unusual modality of monoallelic OR gene expression. We observed substantial variability in the configurations of M71 glomeruli in 88 bulbs examined of 47 conditional *Nrp1* knockout mice, with ectopic M71 glomeruli forming anteriorly and dorsally. We then reassessed the Nrp1 model by analyzing the publicly available mouse strains with which it was formulated originally (Imai et al., 2009). Surprisingly, we could not confirm the simple anterior shift of the medial glomerulus that was reported in the conditional *Nrp1* knockout. The lateral glomerulus typically undergoes a ventral shift and less of an anterior shift. Both medially and laterally, more than one glomerulus is present in the conditional *Nrp1* knockout. The domain of the bulb that these multiple glomeruli occupy can be described as a sector (medially) or belt (laterally). Taken together, our results pose a challenge to the Nrp1 model (Imai et al., 2006, 2009; Luo, 2015). A revision of this model becomes imperative.

## Materials and Methods

### Generation of a gene-targeted mouse strain carrying a *Nrp1* floxed allele

We constructed a targeting vector by long-range PCR to generate a conditional mutation in the *Nrp1* gene by flanking exon 2 with *loxP* sites. The linearized targeting vector was electroporated in the parental embryonic stem cell line E14 of the 129P2/OlaHsd genetic background. G418-resistant clones were screened for homologous recombination by Southern blot hybridization of genomic DNA using an external probe. The FRT-flanked *neo* selectable marker was excised by Flp-mediated recombination. Our *Nrp1* floxed strain is publicly available from The Jackson Laboratory (Bar Harbor, ME) as #6707, official strain name STOCK Nrp1<tm1.1Mom>/MomJ. The design of this targeted mutation is similar to the published *Nrp1* floxed strain (Gu et al., 2003); in both strains, exon 2 of *Nrp1* is excised upon Cre recombination.

### Mouse strains from other laboratories

The Cre reporter strain R26-tauGFP41, carrying the gene-targeted mutation ROSA26-CAGS-τGFP (Mayer et al., 2010) with official allele name Gt(ROSA)26Sor<tm1(CAG-Mapt/GFP)Uboe>, was a generous gift from Uli Boehm (University of Saarland, Homburg, Germany). The gene-targeted *Nrp1* floxed strain (Gu et al., 2003) was obtained from The Jackson Laboratory as #5247, official strain name B6.129(SJL)-Nrp1<tm2Ddg>/J. The transgenic strain I7-Cre-YFP Tg, also referred to as I7-ires-Cre, I7(WT)-Cre, or I7-ires-Cre-ires-gap-YFP (Imai et al., 2009), was obtained from the RIKEN BRC (National Bio-Resource Project of the MEXT, Japan) as #RBRC02932, official strain name C57BL/6-Tg(Olfr16-Olr226,-Cre,-EYFP)1Hsak.

### Mouse husbandry and experimentation

Mice were maintained in specific pathogen–free conditions in individually ventilated cages of the Tecniplast green line. Mice received *ad libitum* γ-irradiated ssniff V1124-727 feed (ssniff, Soest, Germany). Nesting, bedding, and enrichment were provided as nestpak, Datesand Grade 6 (Datesand, Manchester, UK). Mouse experiments were carried out in accordance with guidelines of the National Institutes of Health and the German Animal Welfare Act, European Communities Council Directive 2010/63/EU, and the institutional ethical and animal welfare guidelines of the Max Planck Institute of Biophysics and the Max Planck Research Unit for Neurogenetics. Approval came from the IACUC of The Rockefeller University (New York, NY); the *Regierungspräsidium* Darmstadt (Germany); and the *Veterinäramt* of the City of Frankfurt (Germany).

### Immunohistochemistry on sections

Mice (females and males) were anesthetized by intraperitoneal injection of ketamine HCl and xylazine (210 and 10 mg/kg body weight, respectively), and perfused intracardially with ice-cold PBS followed by 4% paraformaldehyde in PBS. The brain was immersed in 15% sucrose in PBS and 30% sucrose in PBS, each overnight at 4ºC on a shaker. After cryoprotection, the brain was trimmed and frozen in optimal cutting temperature compound (Tissue-Tek) on dry ice in ethanol. Serial horizontal sections encompassing the olfactory bulbs were generated with a Leica CM3050 S cryostat, set at 12-µm thickness. The sections were washed with 1× PBS and blocked with 10% normal donkey serum and 0.3% Triton X-100 in 1× PBS for 1 h at room temperature. After the blocking step, sections were incubated in 3% bovine serum albumin and 0.3% Triton X-100 in 1× PBS overnight at 4˚C with primary antibodies: rabbit anti-Adcy3 (#sc-588, 1:1000; Santa Cruz Biotechnology, Dallas, TX) and goat anti-Nrp1 (#AF566, 1:100; R&D Systems, Minneapolis, MN). Sections were then incubated for 1.5 h at room temperature with secondary antibodies: donkey anti-rabbit IgG Alexa Fluor 647 (#711-606-152, 1:500; Jackson ImmunoResearch Laboratories, West Grove, PA) for rabbit anti-Adcy3, and donkey anti-goat IgG Alexa Fluor 546 (#A11056, 1:1000; Thermo Fischer Scientific, Waltham, MA) for goat anti-Nrp1. Nuclear staining was done with 4′,6-diamidino-2-phenylindole (DAPI; #D1306, 1:10,000; Thermo Fisher Scientific) after the washing steps. Sections were imaged with a Zeiss LSM 710 confocal microscope (Oberkochen, Germany).

### Immunolabeling of whole mounts

Samples were processed according to the iDISCO protocol (Renier et al., 2014). Primary antibodies were goat anti-Nrp1 (#AF566; R&D Systems) and guinea pig anti-VGLUT2 (#135 404; Synaptic Systems, Göttingen, Germany). Secondary antibodies were donkey anti-goat Alexa Fluor 488 (#A11055; Thermo Fisher Scientific) and donkey anti-guinea pig RRX (#706-295-148; Jackson ImmunoResearch Laboratories).

### Serial block-face two-photon tomography

Serial block-face two-photon tomography was performed with a TissueCyte 1000 scanner (TissueVision, Cambridge, MA) equipped with a Zeiss 20× 1.0-NA objective and a Ti:Sapphire laser (Mai Tai HP DeepSee; Spectra-Physics, Santa Clara, CA). Methods were as described in Zapiec and Mombaerts (2015). Briefly, mechanical section thickness was set at 100 µm. Optical *z*-stacks were captured at an *x*,*y* resolution of 1.02 µm per pixel and 5 µm per *z*-plane, resulting in voxels of ∼5 µm^3^. Stacks were captured starting 50 µm below the cutting plane until a depth of 150 µm, thus spanning 100 µm in the *z*-axis. Stacks of images were processed using custom Python, Matlab, and ImageJ scripts provided by TissueVision. The 3D reconstruction and measurements were performed with Amira 6 (FEI, Hillsboro, OR).

## Results

### Glomeruli formed by the coalescence of axons of OSNs that express the OR gene *M71*

The subject of our first study with a conditional *Nrp1* knockout is the population of OSNs that express the OR gene *M71* (M71+ OSNs), also known as *Olfr151*. The choice of this OR gene is motivated by extensive knowledge that has been collected over the past 15 years, including the characterization of odorous ligands for M71 (Bozza et al., 2002). We have reported 39 strains with distinct gene-targeted mutations at the *M71* locus. Importantly, the strain M71-IRES-Cre (Li et al., 2004) is the only gene-targeted Cre driver strain for a mouse OR gene that is publicly available.

**Figure 1.**
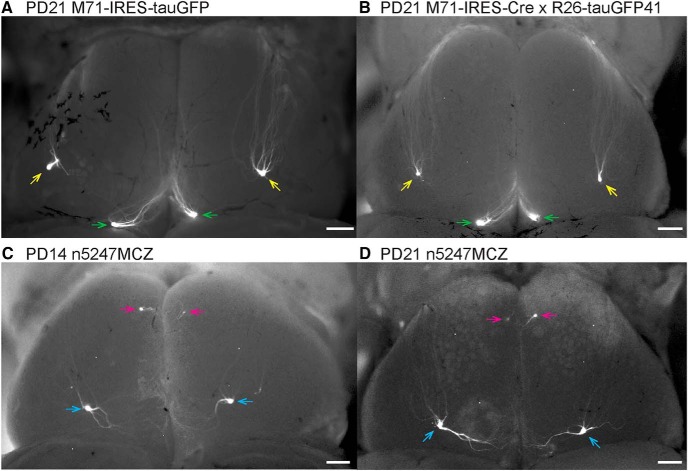
Epifluorescence whole-mount images of bulbs of gene-targeted mice expressing M71 and GFP with and without Nrp1. Images of dorsal views of both bulbs were taken with a Nikon SMZ25 stereomicroscope. Signals represent the intrinsic fluorescence of GFP. Anterior is up, posterior is down, left is left, and right is right. ***A***, Mouse homozygous for M71-IRES-tauGFP at PD21. ***B***, Mouse homozygous for M71-IRES-Cre and homozygous for the R26-tauGFP41 Cre reporter at PD21. ***C***, Triple-mutant n5247MCZ mouse at PD14. ***D***, Triple-mutant n5247MCZ mouse at PD21. Medial and lateral M71 glomeruli at the endogenous positions are indicated with green and yellow arrows, respectively. Ectopic glomeruli in the conditional *Nrp1* knockout are indicated with pink arrows (ectopic anterior) and blue arrows (ectopic dorsal). Scale bars, 100 µm.

M71 is expressed in a few thousand OSNs at PD21 (Bressel et al., 2016). In mice that carry the gene-targeted M71-IRES-tauGFP mutation (Potter et al., 2001), M71^+^ OSNs express tauGFP along with M71 from bicistronic transcripts. The green fluorescent protein–positive (GFP^+^) axons typically coalesce into a single glomerulus (sometimes into two or three glomeruli) posteriorly each in the medial and lateral aspects of the olfactory bulb at PD21 (Fig. 1*A*). The positions of the M71 glomeruli are conserved. Crossing the M71-IRES-Cre strain to the Cre reporter strain R26-tauGFP41 (Mayer et al., 2010; Wen et al., 2011) phenocopies the configuration of labeled glomeruli seen in the M71-IRES-tauGFP strain (Fig. 1*B*). Consistent with their posterior position in the bulb, M71 glomeruli are Nrp1^+^ (Dibattista and Reisert, 2016).

Thus, the population of M71^+^ OSNs represents a convenient and robust model system served by numerous publicly available gene-targeted strains, to assess the role of Nrp1 in determining the positioning of M71 glomeruli along an anterior-posterior axis.

### Ectopic anterior and ectopic dorsal glomeruli in triple-mutant n5247MCZ mice

We determined the effects of a conditional *Nrp1* knockout in M71^+^ OSNs using the same *Nrp1* floxed allele (Gu et al., 2003) that was used in Imai et al. (2009). We refer to this allele as n5247, reflecting the strain number in the catalog of The Jackson Laboratory, from which this strain is publicly available. By repeated crossing, we generated triple-mutant n5247MCZ mice. These mice are homozygous for the *Nrp1* floxed allele (n5247), homozygous for the gene-targeted M71-IRES-Cre allele (MC) (Li et al., 2004), and hemizygous or homozygous for the widely used Cre reporter Z/EG transgene (Z) Novak et al., 2000).

In triple-mutant n5247MCZ mice, M71^+^ OSNs express Cre and become devoid of Nrp1; moreover, they become GFP^+^ by virtue of Cre-mediated excision of a *loxP*-flanked segment of the reporter transgene that results in permanent GFP expression. Examples of whole mounts of bulbs of triple-mutant n5247MCZ mice are shown for PD14 and PD21 (Fig. 1*C*, *D*): a single small GFP^+^ glomerulus is present anteriorly, and a single large GFP^+^ glomerulus, dorsally and posteriorly. Henceforward we refer to these glomeruli as “ectopic anterior” and “ectopic dorsal.” This configuration deviates drastically from the typical configuration of one or a few M71 glomeruli posteriorly in each of the medial and lateral aspects of the bulb (Fig. 1*A*, *B*).

**Figure 2.**
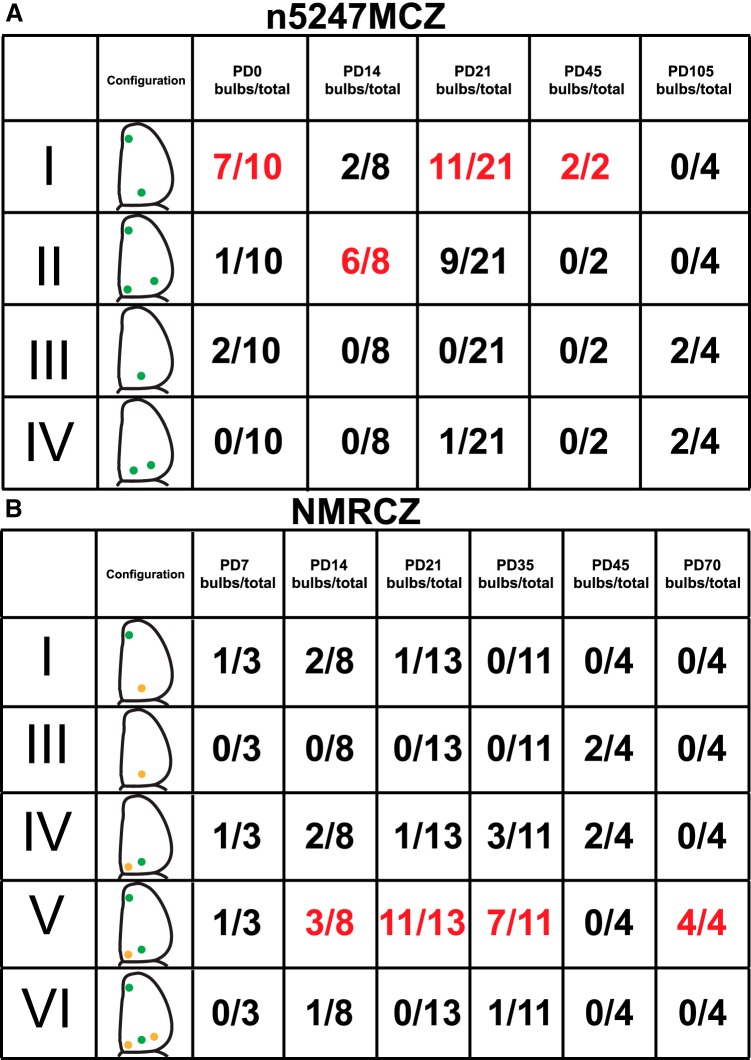
Configurations of M71 glomeruli in bulbs of conditional *Nrp1* knockout mice. ***A***, Triple-mutant mice n5247MCZ. ***B***, Quadruple-mutant mice NMRCZ. The occurrence of configurations I–VI is listed per age. Red numbers indicate the most frequent configuration for a given age. Positions of labeled glomeruli in the various configurations are illustrated schematically with green dots, and in the case of NMRCZ, with orange dots for glomeruli that consist clearly of both RFP^+^ and GFP^+^ axons, and green dots for glomeruli formed predominantly by GFP^+^ axons. No glomeruli formed by only RFP^+^ axons were observed. The data are pooled from whole-mount images and coronal sections. Pairwise χ^2^ tests with Bonferroni correction for multiple comparisons were performed on successive age pairs for both strains: n5247MCZ, PD0 vs. PD14 (*), PD14 vs. PD21 [not significant (n.s.)], PD21 vs. PD105 (***); NMCZ, PD7 vs. PD14 (n.s.), PD14 vs. PD21 (n.s.), PD21 vs. PD35 (n.s.), PD35 vs. PD45 (n.s.), PD45 vs. PD 70 (*). The pre–multiple-comparison sigma values are designated as *p < 0.05, **p < 0.01, and ***p < 0.001.

There was substantial variability, however, in the configurations of sites of coalescence (glomeruli) of GFP^+^ axons when we examined a total of 45 bulbs of 23 triple-mutant n5247MCZ mice at five ages (Fig. 2*A*): 10 bulbs at PD0 (when the glomeruli are beginning to form), eight at PD14, 21 at PD21, two at PD45, and four at PD105. In an attempt to discern distinct categories, we identified at least four configurations of GFP^+^ glomeruli, which we refer to as I, II, III, and IV. Configuration I (22/45 bulbs, 49%) is the most common: it consists of a single small ectopic anterior glomerulus and a single large ectopic dorsal glomerulus and is exemplified in the bulbs shown in Fig. 1*C*, *D*. Configuration II (16 bulbs, 36%) comprises a single small ectopic anterior glomerulus and two glomeruli posteriorly. Configuration III (four bulbs, 9%) consists of a single large ectopic dorsal glomerulus but no ectopic anterior glomerulus. Configuration IV (three bulbs, 7%) has two dorsal glomeruli posteriorly but no ectopic anterior glomerulus. Bulbs with an ectopic anterior glomerulus (or a developing ectopic anterior glomerulus, at PD0) thus occur in 84% of bulbs (I + II, 38/45), and bulbs with a single large ectopic dorsal glomerulus in 58% of bulbs (I + III, 26/45). An ectopic anterior glomerulus is present in 80% of bulbs (8/10) at PD0. At PD105, the oldest age examined, there are no ectopic anterior glomeruli in any bulb (4/4). For comparison with Imai et al. (2009), who report data about PD14 mice, the configuration that we observe most frequently at PD14 is II (6/8, 75%): a single small ectopic anterior glomerulus and two posterior glomeruli.

We have described a linear relationship between the number of OSNs that express a given OR gene and the total volume of the corresponding glomeruli in the bulbs (Bressel et al., 2016). We found that in n5247MCZ mice at PD22, the ectopic anterior glomeruli contribute 20.0% (SD ± 6.5%) to the total glomerular volume per mouse, using methods outlined in Bressel et al. (2016). It follows that ∼20% of Nrp1^–^ M71^+^ OSNs innervate these ectopic anterior glomeruli.

Thus, the multiple configurations of M71 glomeruli in a conditional *Nrp1* knockout and the substantial variability are not consistent with the Nrp1 model for anterior-posterior patterning of glomeruli (Imai et al., 2006, 2009). This model, which was formulated based on results from mice expressing a rat OR from a transgenic mouse MOR23 promoter, does not apply to the first tested population of OSNs that express a mouse OR from its endogenous locus, *M71*.

### Three-dimensional reconstructions of the bulbs of triple-mutant n5247MCZ mice

**Figure 3.**
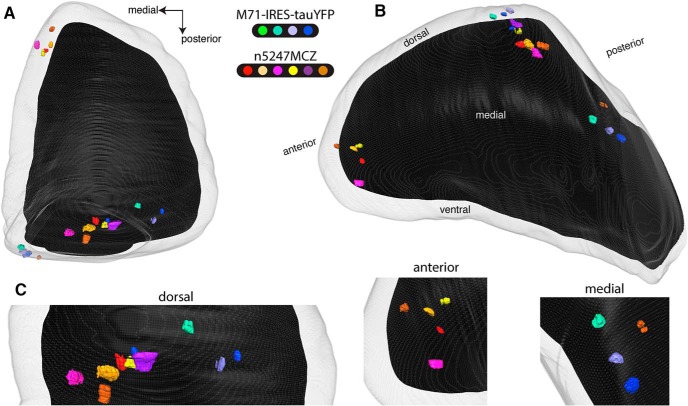
Three-dimensional reconstructions of bulbs of PD21 gene-targeted mice expressing M71 with and without Nrp1. Serial block-face two-photon tomography was carried out to image the intrinsic fluorescence in four bulbs of four homozygous M71-IRES-tauYFP mice at PD21, and in six bulbs of six triple-mutant n5247MCZ mice at PD21. Each individual mouse is indicated with a distinct color. The gray outer area represents the surface of the glomerular layer, and the black inner area the regions of the bulb below the glomerular layer. ***A***, Dorsal view, comparable to the view in Figure 1. The medial M71 glomeruli are poorly visible in this dorsal view, because they reside in a flat, medial domain of the bulb. ***B***, Dorsomedial view. The ectopic anterior glomeruli reside in the rostral tip of the bulb. Both the medial and lateral glomeruli are visible here by making the glomerular layer transparent. The bulb is tilted slightly laterally to expose the medial glomeruli better. ***C***, Close-ups of views oriented in such a way that the individual glomeruli are separated clearly: dorsal, anterior, and medial domains of the bulb.

The variability in configurations of M71 glomeruli in the conditional *Nrp1* knockout poses an unexpected experimental challenge. There are variations on a theme, and further examination may reveal additional configurations or subconfigurations. To enable a direct comparison of the positions of M71 glomeruli with and without Nrp1, we imaged bulbs using TissueCyte serial block-face two-photon tomography (Ragan et al., 2012) and generated three-dimensional (3D) reconstructions of bulbs (Zapiec and Mombaerts, 2015). We made use of the intrinsic fluorescence signal in glomeruli in four bulbs of PD21 mice homozygous for the gene-targeted M71-IRES-tauYFP mutation (Nrp1^+^) and in six bulbs of triple-mutant n5247MCZ mice (Nrp1^–^) also at PD21. Methods of image acquisition, registration, and image processing have been described in detail (Zapiec and Mombaerts, 2015). Figure 3 and Movie 1 show views of the merged right olfactory bulb, which is composed of a total of 10 left or right olfactory bulbs. The views on this merged bulb are, respectively, dorsal and comparable to Figure 1 (Fig. 3*A*), and dorsomedial and slightly tilted laterally (Fig. 3*B*) so that the posterior-medial glomeruli are more visible. Figure 3*C* provides close-ups. All four M71-IRES-tauYFP bulbs have a single medial glomerulus posteriorly; two have a single lateral glomerulus posteriorly, and two have two lateral glomeruli posteriorly. These configurations are consistent with what has been reported for M71 glomeruli in several gene-targeted strains at or around PD21 (for instance, Potter et al., 2001; Feinstein and Mombaerts, 2004; Feinstein et al., 2004; Zou et al., 2004). The configuration of glomeruli in the 3D reconstructions of bulbs of triple-mutant n5247MCZ mice deviates drastically from that in the M71-IRES-tauYFP mice. Four n5247MCZ bulbs show configuration I (Fig. 2*A*), with a single ectopic anterior glomerulus and a single ectopic dorsal glomerulus; this configuration is the most frequently seen at PD21. One n5247MCZ bulb is of configuration II (one ectopic anterior glomerulus and two glomeruli posteriorly), and another n5247MCZ bulb is of configuration III, with a single ectopic dorsal glomerulus. New information from these 3D reconstructions is that the ectopic dorsal glomeruli reside roughly halfway between the medial and lateral M71 glomeruli, and at approximately the same anterior-posterior position.

Movie 1. 3D animation of a merged bulb containing glomeruli from three M71-IRES-tauYFP bulbs and six n5247MCZ bulbs at PD21. The merged bulb 3D reconstruction displays the positions of labeled glomeruli from a total of nine bulbs together, enabling a direct comparison of these positions. This animation provides multiple viewing angles of the merged bulb shown in Figure 3. Glomeruli from M71-IRES-tauYFP bulbs are rendered in cold colors (green/blue) as indicated by the legend in the top left, and glomeruli from n5247MCZ bulbs are in warm colors (pink/orange/purple/red/yellow/brown). The sequence starts with a view of the dorsal aspect of the bulb with the posterior-dorsal glomeruli from M71-IRES-tauYFP bulbs visible near the bottom, and the posterior-medial glomeruli visible in a small cluster near the left of the screen. As the camera pans to reveal the medial aspect of the bulb, the glomeruli from n5247MCZ bulbs are revealed sequentially. The camera then progresses along the medial aspect of the bulb from posterior to anterior, before panning out to stop on a long view of the medial aspect of the bulb. 10.1523/ENEURO.0123-16.2016.video.1

Thus, the 3D reconstructions of bulbs by serial block-face two-photon tomography confirm and extend the observations made by whole-mount epifluorescence. The multiple configurations of M71 glomeruli and the substantial variability are not consistent with the Nrp1 model for anterior-posterior patterning of glomeruli.

### Quadruple-mutant NMRCZ mice

OR genes are expressed monoallelically: an OSN that expresses a given OR gene expresses one allele of it (Chess et al., 1994; Strotmann et al., 2000; Ishii et al., 2001). This monoallelic expression is extremely tight (Saraiva et al., 2015). Next, we developed a novel genetic strategy that capitalizes on this unusual modality of gene expression in the mouse. The quadruple-mutant mice NMRCZ enable a direct, mosaic comparison of Nrp1^+^ and Nrp1^–^ populations of M71^+^ OSNs within the same mouse, as follows.

**Figure 4.**
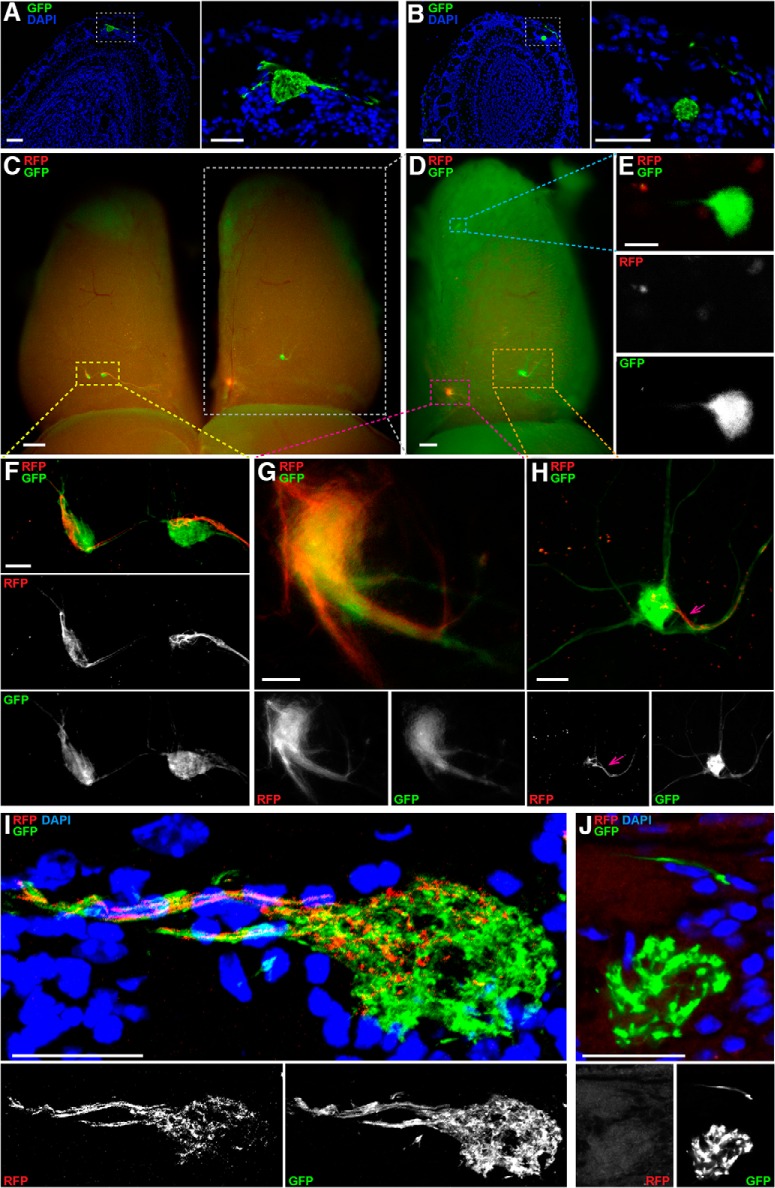
Confocal and whole-mount imaging of M71 glomeruli in triple-mutant NMCZ and quadruple-mutant NMRCZ mice. ***A***, ***B***, Coronal 12-µm sections of bulbs of NMCZ mice at PD21 imaged with a Zeiss LSM 710 confocal microscope, using the intrinsic fluorescence of GFP and counterstaining of nuclei with DAPI. The box indicated with a white stippled line in the left images is magnified in the right images. ***A***, Ectopic dorsal glomerulus, large. ***B***, Ectopic anterior glomerulus, small and located deeper in the glomerular layer. ***C–H***, Whole-mount bulbs of an NMRCZ mouse at PD59 imaged in wide-field on a Nikon SMZ25 stereofluorescence microscope. ***C***, Dorsal view of the left and right bulbs, with the left bulb exhibiting configuration VI, and the right bulb, configuration V. Not all glomeruli are visible in this view. ***D***, The right bulb from ***C*** (white box) is tilted to the right to provide a better view of the medial aspect and demonstrate the configuration consisting of an ectopic anterior glomerulus (blue box), a medial glomerulus (red box), and an ectopic dorsal glomerulus (orange box). ***E***, Magnified view of the ectopic anterior glomerulus and consisting of GFP^+^ axons, as shown in the blue box in ***D***. ***F***, Magnified view of the two dorsal glomeruli of the left bulb, in the yellow box in ***C***. The left glomerulus exhibits a mixed, comparable contribution of GFP^+^ and RFP^+^ axons. The right glomerulus consists mostly of GFP^+^ axons, with a compartment of RFP+ axons. ***G***, Magnified view of the mixed GFP^+^ RFP^+^ medial glomerulus of the right bulb, as shown in the red box in ***D***. ***H***, Magnified view of the ectopic dorsal glomerulus consisting predominantly of GFP^+^ axons, as shown in the orange box in ***D***. A small compartment of this glomerulus is innervated by an axon bundle containing also RFP^+^ axons (pink arrow). ***I, J,*** 12-µm sections of bulbs of NMRCZ mice at PD56 and PD70, respectively, imaged with a Zeiss LSM 710 confocal microscope, using the intrinsic fluorescence of GFP and RFP and counterstaining of nuclei with DAPI. ***I***, Ectopic dorsal glomerulus consisting of RFP^+^ and GFP^+^ axons. ***J***, Magnified view of an ectopic-anterior glomerulus consisting of GFP^+^ axons. Scale bars, ***A*** left, 100 µm; ***A*** right, 50 µm; ***B*** left, 100 µm; ***B*** right, 50 µm; ***C*** and ***D***, 300 µm; ***E***, 20 µm; ***F***, 50 µm; ***G***, 25 µm; ***H***, 50 µm; and ***I*** and ***J***, 25 µm.

We generated a novel floxed allele of *Nrp1* by flanking exon 2 with *loxP* sites, adopting a similar design as for the published *Nrp1* floxed allele (Gu et al., 2003). We then produced two triple-mutant strains by repeated crossing: NMCZ and NMRZ. The triple-mutant strain NMCZ is homozygous for our *Nrp1* floxed allele (N), homozygous for M71-IRES-Cre (MC), and hemizygous or homozygous for the Z/EG transgene (Z); it is analogous to the triple-mutant n5247MCZ strain but with our *Nrp1* floxed allele. As in triple-mutant n5247MCZ mice, ectopic glomeruli also exist in triple-mutant NMCZ mice, both the large ectopic dorsal glomerulus (Fig. 4*A*) and the smaller ectopic anterior glomerulus (Fig. 4*B*). The triple-mutant strain NMRZ is homozygous for our *Nrp1* floxed allele (N), homozygous for M71-IRES-tauRFP2 (MR) (Li et al., 2004), and hemizygous or homozygous for the Z/EG transgene (Z). By crossing an NMCZ mouse with an NMRZ mouse, quadruple-mutant NMRCZ offspring are obtained that are homozygous for our *Nrp1* floxed allele (N), compound heterozygous at the *M71* locus (M71-IRES-Cre and M71-IRES-tauRFP2; MRC), and hemizygous or homozygous for the Z/EG reporter (Z). In these quadruple-mutant mice, OSNs that express the M71-IRES-tauRFP2 allele are red fluorescent protein–positive (RFP^+^) and Nrp1^+^, and OSNs that express the M71-IRES-Cre allele are GFP^+^ (via expression of the Z/EG reporter) and Nrp1^–^.

We analyzed a total of 43 bulbs of 24 quadruple-mutant NMRCZ mice at six ages: three at PD7, eight at PD14, 13 at PD21, 11 at PD35, four at PD45, and four at PD70 (Fig. 2*B*). There are no bulbs of configuration II, which we had identified earlier in triple-mutant n5247MCZ mice. In addition to the configurations I (four bulbs, 9%), III (two bulbs, 5%), and IV (nine bulbs, 21%), we identified two new configurations in quadruple-mutant NMRCZ mice. In configuration V (Fig. 4*C*, right, and *D*), the most frequent configuration (26 bulbs, 60%), there is a single small ectopic anterior glomerulus anteriorly (Fig. 4*E*, *J*), a single medial glomerulus (Fig. 4*G*), and a single large ectopic dorsal glomerulus (Fig. 4*H*). The medial glomerulus is innervated clearly in a mixed fashion, but to various extents, by RFP^+^ axons and GFP^+^ axons. The position of this medial glomerulus is typical for the medial M71 glomerulus. Configuration VI (two bulbs, 4%) has the same glomeruli as configuration V plus a single lateral glomerulus, at a position that is typical for the lateral M71 glomerulus (Fig. 4*C*, left, and *F*). Altogether, in 65% of bulbs (V + VI, 28/43), the RFP^+^ axons (representing the M71-IRES-tauRFP2 allele, without Cre recombination) coalesce into glomeruli at positions that are typical for M71 glomeruli, and they mix in these glomeruli with GFP^+^ axons (representing the M71-IRES-Cre allele, with Cre recombination; Fig. 4*I*).

**Figure 5.**
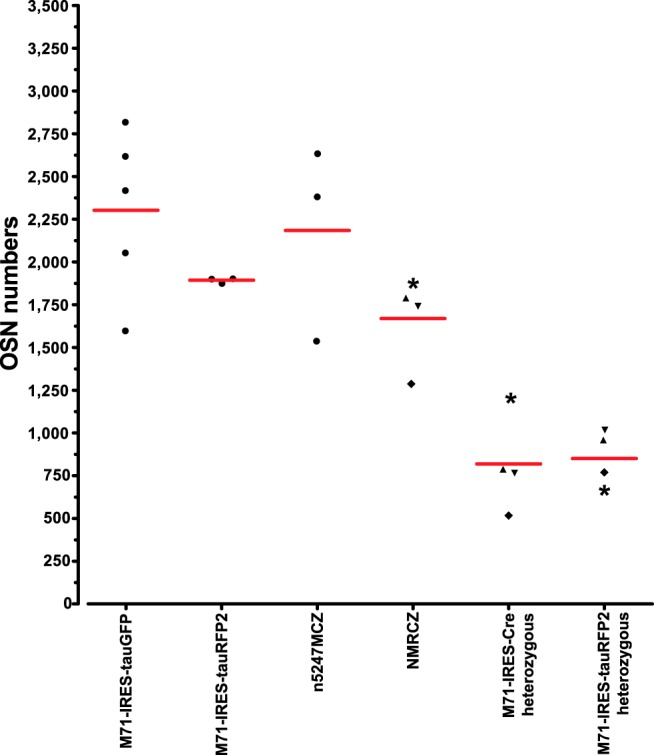
Numbers of labeled cells per mouse in mice with gene-targeted mutations in the *M71* locus at PD21. Numbers are given ± SD and are Abercrombie corrected, as described in Bressel et al. (2016). A symbol represents an individual mouse. The numbers for M71-IRES-tauGFP (*n* = 5 mice) and M71-IRES-tauRFP2 (*n* = 3 mice) are taken from Bressel et al. (2016) for comparison. “M71-IRES-Cre heterozygous” and “M71-IRES-tauRFP2 heterozygous” are the numbers of labeled cells representing the two different M71 alleles in NMRCZ (*n* = 4 mice), and the sum of these numbers is given in “NMRCZ.” The symbols (asterisk, upward triangle, downward triangle, diamond) used for NMRCZ represent individual mice. Analysis of cell counts using one-way ANOVA found all homozygous vs. heterozygous pairs significant (*p* < 0.05) according to Tukey’s multiple comparison test, which is consistent with monoallelic expression of OR genes. All other comparisons (–/– vs. –/– pairs and +/– vs. +/– pairs) are not significant.

The numbers of labeled OSNs in the triple-mutant and quadruple-mutant mice are comparable to those in M71-IRES-tauGFP mice at PD21 (Fig. 5): n5247MCZ *t* test *p* = 0.7635, NMRCZ *t* test *p* = 0.0526. These numbers were determined with Abercrombie correction, as detailed in Bressel et al. (2016). We did not find a significant difference between the numbers of labeled OSNs expressing the M71-IRES-Cre or M71-IRES-tauRFP2 allele in NMRCZ mice (*t* test *p* = 0.85).

### Glomeruli in posterior domains of the bulb can be Nrp1^+^ or Nrp1^–^

Nrp1 expression in OSNs is dependent on expression of the cAMP-generating enzyme adenylate cyclase Adcy3 (Dal Col et al., 2007). Most if not all mature OSNs express Adcy3. An exception is a population of chemosensory neurons in the mouse main olfactory epithelium that express the cation channel Trpc2 (Liman et al., 1999), the cyclic-nucleotide gated channel subunit Cnga2, and the guanylate cyclase Gucy1b2: they do not express Adcy3 (Omura and Mombaerts, 2014, 2015). Glomeruli of Gucy1b2^+^ neurons can be examined easily and specifically in mice of the gene-targeted Gucy1b2-IRES-tauGFP strain: neurons that express Gucy1b2 coexpress GFP, and the expression level is high enough to visualize the signal in glomeruli by the intrinsic fluorescence of GFP (Omura and Mombaerts, 2015). Axons of Gucy1b2^+^ neurons coalesce typically into three glomeruli posteriorly in the olfactory bulb (Omura and Mombaerts, 2015). The absence of Adcy3 expression in Gucy1b2^+^ neurons and the morphological similarity between Gucy1b2^+^ glomeruli and canonical glomeruli formed by the coalescence of axons of OR-expressing OSNs predict that Gucy1b2 glomeruli are Nrp1^–^.

**Figure 6.**
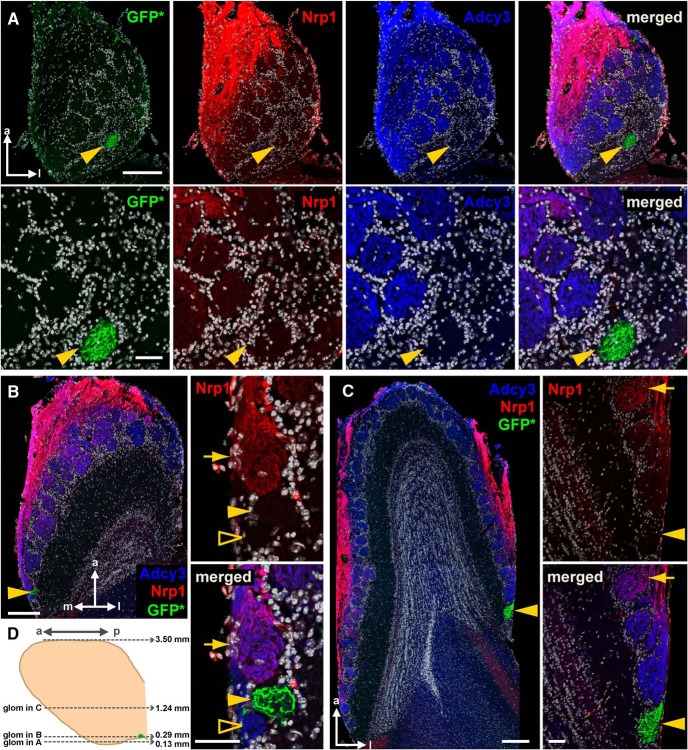
Posterior glomeruli in the vicinity of Gucy1b2^+^ glomeruli can be Nrp1^+^ or Nrp1^–^. Fluorescence images of three horizontal sections of a bulb of a homozygous Gucy1b2-IRES-tauGFP mouse at 4 weeks were taken with a Zeiss LSM 710 confocal microscope. Each of the three Gucy1b2^+^ glomeruli is indicated with a yellow arrowhead. ***A***, Section at a very ventral level, 0.13 mm from the bottom of the bulb, as shown schematically in ***D***. Intrinsic GFP fluorescence (GFP*) is combined with immunofluorescence for Nrp1 (red) and Adcy3 (blue). Nuclear staining with DAPI is in white. Merged is all colors together. The top four panels show the entire section. The bottom four panels show high-magnification views of an area surrounding the GFP^+^ glomerulus. This glomerulus is Adcy3^–^ and Nrp1^–^. The posterior part of the bulb at this very ventral level is devoid of Nrp1 immunofluorescence but contains numerous glomeruli that are Adcy3^+^. ***B***, Section at a more dorsal level, 0.29 mm from the bottom of the bulb. The image on the left shows the entire section. The two panels on the right show high-magnification views of an area surrounding the GFP^+^ glomerulus. This extremely posterior GFP^+^ glomerulus is Adcy3^–^ and Nrp1^–^. The glomerulus anterior to the GFP^+^ glomerulus is Adcy3^+^ and Nrp1^+^ (yellow arrow), but the glomerulus posterior to the GFP^+^ glomerulus is Adcy3^+^ and Nrp1^–^ (unfilled yellow arrowhead). ***C***, Section at an intermediate dorsal-ventral level, 1.24 mm from the bottom of the bulb. The image on the left shows the entire section. The two panels on the right show high-magnification views of an area surrounding the GFP^+^ glomerulus. This very posterior GFP^+^ glomerulus is Adcy3^–^ and Nrp1^–^. The glomeruli anterior to it are strongly Adcy3^+^ and Nrp1^–^, and the most anterior glomerulus (indicated with an arrow) is Adcy3^+^ and weakly Nrp1^+^. ***D***, Schematic diagram of a medial view on the bulb. The positions of the three glomeruli shown in ***A–C*** are indicated. Scale bars, ***A***, top 200 μm, bottom 50 μm; ***B*** and ***C***, left 200 μm, right 50 μm. a, anterior; p, posterior; m, medial; l, lateral; glom, glomerulus.

On a horizontal section at an extremely ventral level through the bulb of a homozygous Gucy1b2-IRES-tauGFP mouse at 4 weeks, a single GFP^+^ glomerulus could be observed at a lateral position (Fig. 6*A*). This glomerulus was Adcy3^–^ and Nrp1^–^. Interestingly, numerous glomeruli anterior to this GFP^+^ glomerulus were Adcy3^+^ but Nrp1^–^, thus breaking the causal link between Adcy3 and Nrp1 expression. On a horizontal section at a slightly more dorsal level, another GFP^+^ glomerulus at a medial position was also Adcy3^–^ and Nrp1^–^, and adjacent glomeruli were Adcy3^+^ but either Nrp1^+^ or Nrp1^–^ (Fig. 6*B*). At an intermediate dorsal-ventral level of this bulb, a third GFP^+^ glomerulus, at a lateral position, was Adcy3^–^ and Nrp1^–^, but the glomeruli just anterior to it were Adcy3^+^ and Nrp1^–^ or very weakly Nrp1^+^ (Fig. 6*C*).

Thus, by examining three posterior domains of a bulb that are defined by Gucy1b2^+^ glomeruli, we found that there is no absolute link between Adcy3 and Nrp1 expression, and that posterior glomeruli can be either Nrp1^+^ or Nrp1^–^.

### Nrp1 levels in a 3D reconstruction of a bulb of a wild-type C57BL6/J mouse

It thus became necessary to take a bulb-wide view of Nrp1 levels, and to revisit the proposed anterior-posterior Nrp1 gradient (Imai et al., 2006, 2009), at the level of the entire bulb and in 3D (Zapiec and Mombaerts, 2015).

**Figure 7.**
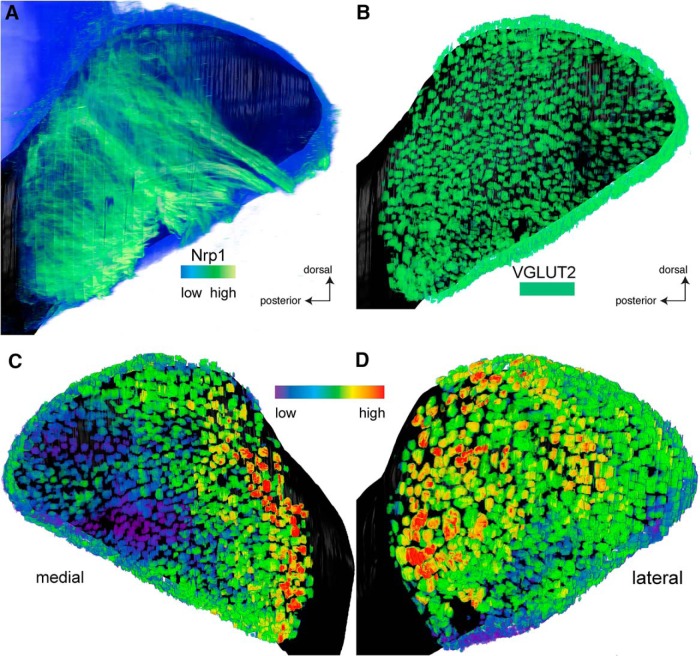
Three-dimensional reconstructions of bulbs with Nrp1 immunofluorescence levels in glomeruli of a bulb of a wild-type C57BL6/J mouse at PD14. After iDISCO treatment of the bulb, immunofluorescence with antibodies against Nrp1 and VGLUT2 was applied to the whole mount, followed by serial block-face two-photon tomography and 3D reconstruction. ***A***, Overall Nrp1 immunofluorescence signal, medial view on the bulb. The lookup table from low (blue) to high (green) represents the intensity of the overall Nrp1 immunofluorescence signal, which is much stronger in axons than in glomeruli. The signal in glomeruli is mostly obscured by the signal in overlying axons. ***B***, Segmented VGLUT2 immunofluorescence signal, shown in uniform green, binary signal, with no intensity information. VGLUT2 is a marker for OSN axon terminals within glomeruli. The layers of the bulb below the glomerular layer are rendered in black for contrast and orientation. ***C***, Medial view of the reconstructed bulb. The Nrp1 signal is confined to glomeruli by generating a mask using the VGLUT2 signal. The lookup table from low (violet) to high (red) represents the intensity of the Nrp1 immunofluorescence signal in glomeruli. Generally, anterior glomeruli are Nrp1^low^ (from violet to blue), and generally, posterior glomeruli are Nrp1^high^ (from green over yellow to red). ***D***, Lateral view of the reconstructed bulb. Generally, anterior glomeruli are Nrp1^low^, and generally, posterior glomeruli are Nrp1^high^.

We performed whole-mount Nrp1 immunofluorescence of wild-type C57BL/6J bulbs at PD14 using the iDISCO protocol (Renier et al., 2014), and imaged and reconstructed the labeled bulbs by applying serial block-face two-photon tomography. Figure 7 shows a representative bulb. The Nrp1 antibody labels glomeruli efficiently, but it also labels OSN axons in the olfactory nerve layer (Fig. 7*A*), thus obscuring the glomeruli in 3D reconstructions of the bulb. Although we could assess the Nrp1 levels within individual glomeruli when examining the tomography sections, we wanted to obtain a global view of the 3D reconstructed bulb, which required segmenting the glomerular layer from the olfactory nerve layer. This segmentation was accomplished by colabeling with an antibody for VGLUT2, a robust marker of glomeruli that labels OSN axon terminals within glomeruli (Richard et al., 2010). We used the VGLUT2 signal to define the glomeruli by thresholding this signal to perform semi-automated segmentation. By masking the Nrp1 signal to display only the voxels that belong to glomeruli as defined by the VGLUT2 signal (Fig. 7*B*), we visualized the intensity of the Nrp1 immunofluorescence signal specifically in glomeruli, and thereby obtained clear views of Nrp1 levels in glomeruli across the 3D reconstructed bulb.

A medial view (Fig. 7*C*) and a lateral view (Fig. 7*D*) of the 3D reconstruction reveal that, generally, Nrp1^low^ glomeruli reside anteriorly and dorsally, and Nrp1^high^ glomeruli posteriorly. But upon closer examination, the pattern of Nrp1 levels is mosaic and patchy: there is considerable variability in staining intensity among neighboring glomeruli. Nrp1^low^ glomeruli can be found in the immediate vicinity of Nrp1^high^ glomeruli, as we had observed in the three posterior domains defined by Gucy1b2^+^ glomeruli. The olfactory bulb is often described as comprising two mirror-image maps of glomeruli (two half-bulbs; Nagao et al., 2000) such that glomeruli for a given OR can be found in positions reflected along a mirror plane. But no such symmetry was obvious when we examined the patterns of Nrp1 immunofluorescence signal intensities among the medial and lateral aspects of the bulb.

Further quantitative analyses will be required to settle definitively the hypothesis of a smooth anterior-posterior bulb-wide Nrp1 gradient, and across various possible anterior-posterior “axes.” Several pre- and postnatal ages must also be examined.

### Glomeruli formed by the coalescence of axons of OSNs that express rat OR I7 from a transgenic mouse MOR23 promoter with and without Nrp1

**Figure 8.**
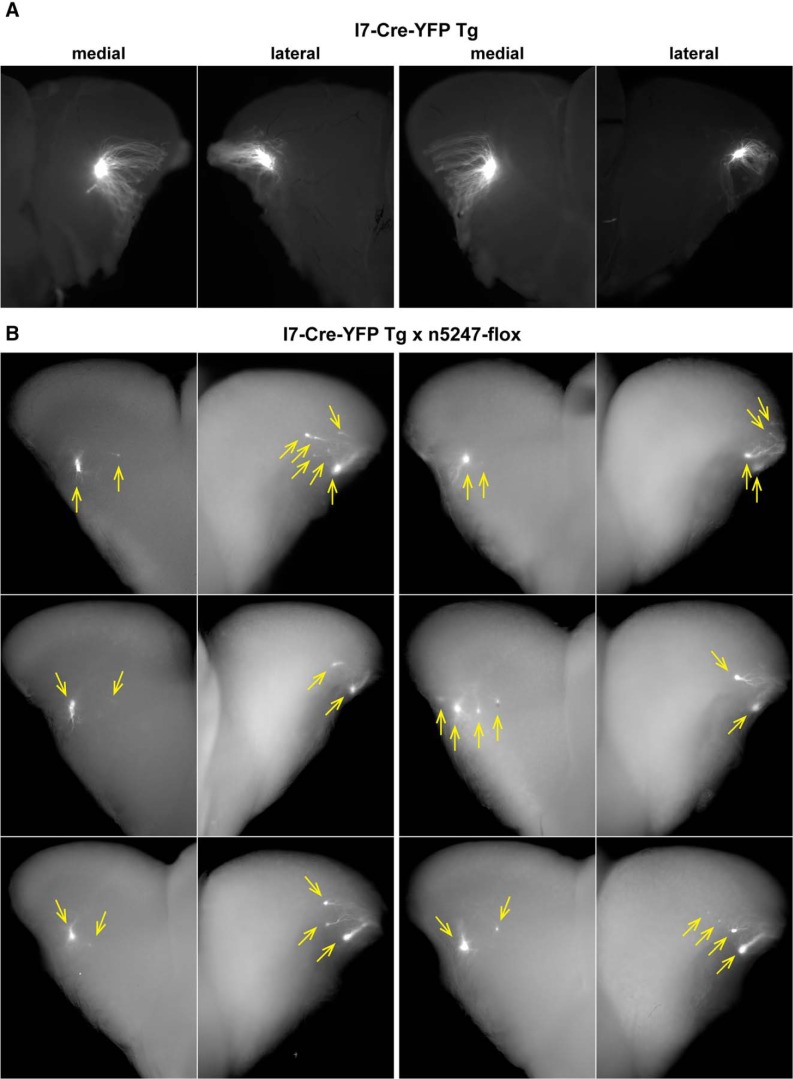
Epifluorescence whole-mount images of bulbs of transgenic mice expressing rat OR I7 from a mouse MOR23 promoter and gap-YFP with and without Nrp1. Images of medial and lateral views of bulbs were taken with a Nikon SMZ25 stereomicroscope. Signal represents the intrinsic fluorescence of YFP. Dorsal is up, ventral is down. ***A***, Views on the medial and lateral aspects of a left bulb (left two images) and a right bulb (right two images) of two I7-Cre-YFP Tg mice at PD17. Axons coalesce into a single glomerulus. ***B***, Views on the medial and lateral aspects of the right bulbs of six I7-Cre-YFP Tg × n5247-flox littermates at PD14. Images are pairwise for an individual mouse. Glomeruli are indicated with yellow arrows. Axons coalesce into multiple glomeruli, in particular in the lateral aspect.

We sought to replicate the experiments with the transgenic mice that express the rat OR I7 from a mouse MOR23 promoter along with the Cre recombinase and gap-YFP (Imai et al., 2009). This mouse strain is publicly available from the RIKEN BioResource Center and is here referred to as I7-Cre-YFP Tg. In the gap-YFP marker, the 20 N-terminal amino acid residues of GAP43 are fused to the N terminus of the yellow fluorescent protein (YFP) to target it to the plasma membrane (Moriyoshi et al., 1996). Figure 8*A* shows epifluorescence whole-mount images of the medial and lateral aspects of a left and a right bulb of two I7-Cre-YFP Tg mice at PD17, visualizing the intrinsic fluorescence from the gap-YFP fusion protein. We confirm and extend the observation (Imai et al., 2009) that axons expressing rat OR I7 from this transgenic mouse MOR23 promoter coalesce into a single glomerulus in the medial aspect of the bulb, and add that a single labeled glomerulus is present in the lateral aspect of the bulb.

We then crossed I7-Cre-YFP-Tg mice to gene-targeted mice carrying the *Nrp1* floxed allele n5247 (Gu et al., 2003), thus reproducing the same cross used in Figure 1*A* of Imai et al. (2009). Figure 8*B* shows epifluorescence whole-mount images of the medial and lateral aspects of six bulbs of six I7-Cre-YFP Tg littermates at PD14, the same age analyzed in Imai et al. (2009). We made distinct observations for the medial and lateral aspects of these bulbs. Medially, we observed two labeled glomeruli in five of the six bulbs, and four labeled glomeruli in one bulb. One of these glomeruli appeared larger than the others, and was more anterior except in the case of the four labeled glomeruli, of which the largest was the second most anterior glomerulus. A virtual line drawn between the labeled glomeruli in a given bulb yielded various orientations or axes, which were grossly along the anterior-posterior dimension but also had a ventral or dorsal component. Laterally, we observed two to six labeled glomeruli (average 3.5), with one glomerulus larger than the others. Connecting the labeled glomeruli in a given bulb did not reveal a consistent orientation or axis. Because there is no information about the labeled glomeruli in the lateral aspect of the bulb in Imai et al. (2009), a comparison of the data is not possible.

Thus, the positions of the labeled glomeruli and their substantial variability in the I7-Cre-YFP Tg × n5247-flox cross are not consistent with the Nrp1 model for anterior-posterior patterning of glomeruli for this novel, artificial population of OSNs that express rat OR I7 from a transgenic mouse MOR23 promoter.

### 3D reconstructions of the bulbs of I7-Cre-YFP Tg mice with and without Nrp1

Finally, as we had done for triple-mutant n5247MCZ mice, we imaged labeled bulbs of I7-Cre-YFP Tg mice with and without Nrp1, by applying serial block-face two-photon tomography based on the intrinsic fluorescence of the gap-YFP marker and then generating 3D reconstructions of the bulbs.

**Figure 9.**
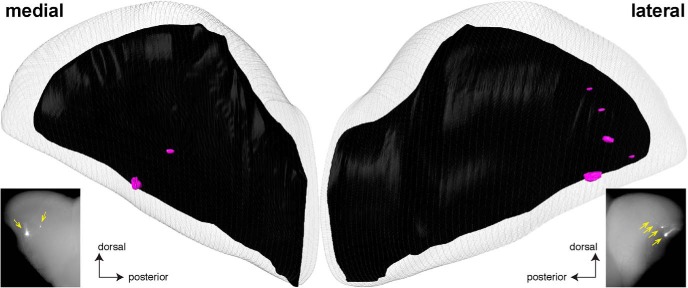
Example of a three-dimensional reconstruction of a bulb of a PD14 transgenic mouse expressing rat OR I7 from a mouse MOR23 promoter and gap-YFP. The right bulb of a mouse was reconstructed in 3D after epifluorescence whole-mount imaging with a Nikon SMZ25 stereomicroscope. The images of the medial and lateral aspects of this bulb are the same as in Figure 8, right bottom, and reproduced here to facilitate comparison with the 3D reconstruction. There are two labeled glomeruli in the medial aspect (compared to two in the epifluorescence whole-mount image, yellow arrows), and five labeled glomeruli in the lateral aspect (compared to four in the epifluorescence whole-mount image, yellow arrows).

Figure 9 shows one example of a reconstructed (right) bulb of a I7-Cre-YFP Tg mouse at PD14; it is the same bulb as shown in the bottom right panels of Fig. 8*B*. For comparison, the epifluorescence whole-mount images of the same bulb are shown again. The two labeled glomeruli in the medial aspect seen in the epifluorescence whole-mount image are clearly detectable in the 3D reconstruction. In the lateral aspect, a fifth, small labeled glomerulus is detectable in the 3D reconstruction, but is not seen in the epifluorescence whole-mount image.

**Figure 10.**
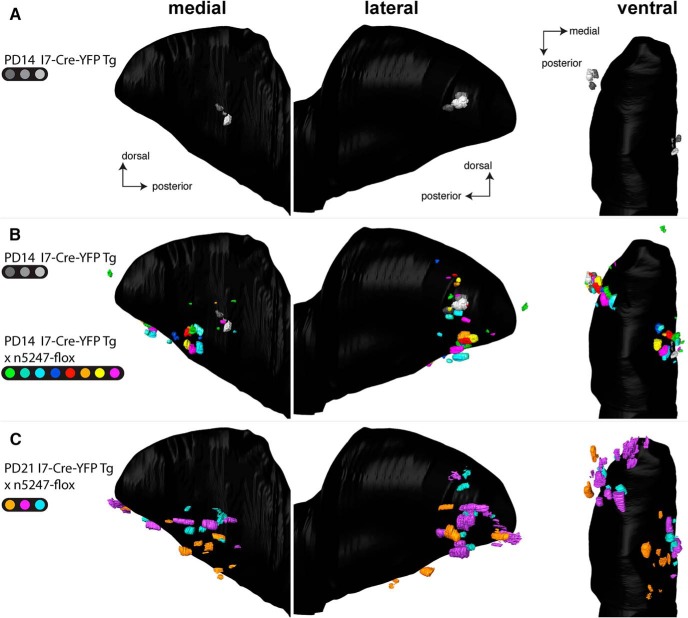
Three-dimensional reconstructions of bulbs of transgenic mice expressing rat OR I7 from a mouse MOR23 promoter and gap-YFP with and without Nrp1. For PD14, three bulbs of three I7-Cre-YFP Tg littermates (indicated in shades of gray) and eight bulbs of seven I7-Cre-YFP Tg × n5247-flox littermates (indicated in distinct colors) were reconstructed. For one mouse, both the left and right bulbs (green and turquoise) are included in the merged bulb. For PD21, three bulbs of three I7-Cre-YFP Tg × n5247-flox littermates were reconstructed. Views are medial, lateral, and ventral. ***A***, Merged bulb of three I7-Cre-YFP Tg bulbs at PD14. ***B***, Merged bulb of three I7-Cre-YFP Tg bulbs at PD14 together with eight I7-Cre-YFP Tg × n5247-flox bulbs at PD14. Because of the large number of glomeruli depicted, the gray-transparent outer layer of the bulb shown in Figures 3 and 9 is here omitted. Medially, I7-Cre-YFP Tg × n5247-flox glomeruli are shifted anteriorly and ventrally and scattered over a sector that is anchored on the tightly clustered I7-Cre-YFP Tg glomeruli. Laterally, I7-Cre-YFP Tg × n5247-flox glomeruli are shifted dorsally and ventrally and scattered over a belt that is centered on the tightly clustered I7-Cre-YFP Tg glomeruli. This belt surrounds the anterior bulb as a U-shape but is not continuous ventrally, as can be seen in the ventral view. ***C***, Merged bulb of three I7-Cre-YFP Tg × n5247-flox bulbs at PD21. These mice also contain the reporter ROSA-STOP-lacZ, but the taulacZ marker is not visualized. The number and degree of scattering of glomeruli is more pronounced than at PD14.

Figure 10*A* shows a merged bulb of three I7-Cre-YFP Tg bulbs at PD14, including the bulb shown in Figure 9. The 3D reconstruction reveals a single labeled glomerulus in each the medial and lateral aspects of the bulb. The advantage of the merged bulb approach is that glomerular positions can be compared directly: we found that the three glomeruli were tightly clustered in each of the medial and lateral aspects. In sharp contrast, the merged bulb composed of eight I7-Cre-YFP Tg × n5247-flox bulbs and the three I7-Cre-YFP Tg bulbs at PD14 (thus 11 bulbs in total) revealed a broad scattering of multiple I7-Cre-YFP Tg × n5247-flox glomeruli in both the medial and lateral aspects of the bulb (Fig. 10*B*). Medially, the scattered glomeruli (mean 2.3, minimum 1, maximum 5) occupied a broad domain of the bulb that can best be characterized as a sector, anchored at the position of the tightly clustered glomeruli of the I7-Cre-YFP Tg bulbs and radiating anteriorly and ventrally. Laterally, the scattered glomeruli (mean 3.0, minimum 2, maximum 5) were centered at the tightly clustered glomeruli of the I7-Cre-YFP Tg bulbs; seven glomeruli underwent a dorsal shift, 14 a ventral shift, and three no shift. A ventral view of this merged bulb (Fig. 10*B*) revealed that the glomeruli occupied a belt that spanned the ventral extent and connected the medial and lateral domains of the I7-Cre-Tg glomeruli with Nrp1. This glomerular belt was interrupted for a segment defined by the ventral ridge of the bulb. In three I7-Cre-YFP Tg × n5247-flox bulbs at PD21, there were more labeled glomeruli (medially: mean 6, minimum 4, maximum 9; laterally: mean 5.7, minimum 3, maximum 8), and the degree of scattering both medially and laterally was increased (Fig. 10*C*). The distribution of glomeruli in the PD14 and PD21 bulbs can be evaluated in Movie 2.

Movie 2. 3D animation of a merged bulb containing glomeruli from three PD14 I7-Cre-YFP-Tg bulbs, eight PD14 I7-Cre-YFP-Tg × n5247-flox bulbs, and three PD21 I7-Cre-YFP-Tg × n5247-flox bulbs. The animation begins with a view of the medial aspect of the merged bulb with anterior located to the left and dorsal on top. Three glomeruli, each from a different PD14 I7-Cre-YFP Tg mouse, are displayed together with the shade of gray indicating the identity. The legends on the top left and bottom left of the screen indicate the strain of all glomeruli shown. As the animation proceeds, glomeruli from PD14 I7-Cre-YFP-Tg × n5247-flox bulbs are added sequentially. These glomeruli occupy a sector of the bulb extending roughly from the medial domain of PD14 I7-Cre-YFP-Tg glomeruli toward and beyond their lateral domain by way of the ventral ridge of the bulb. The ventral view of the bulb shown at the 11-s mark provides a clear view of the belt of glomeruli straddling the ventral aspect of the bulb. Finally, the glomeruli from PD14 bulbs are removed, and glomeruli from three PD21 I7-Cre-YFP-Tg bulbs are shown; these glomeruli occupy a broad sector of both the medial and lateral aspects. 10.1523/ENEURO.0123-16.2016.video.2

Taken together, our observations with the cross I7-Cre-YFP Tg × n5247-flox at the same age of PD14 are not consistent with the data reported in Imai et al. (2009). Our findings do not support the Nrp1 model of anterior-posterior patterning of glomeruli in the bulb for this novel population of OSNs that express rat OR I7 from a transgenic mouse MOR23 promoter.

## Discussion

Naturally, any model that aspires to explain how axons of ∼1100 populations of OSNs, each expressing a distinct OR gene sort and coalesce into ∼3600 glomeruli at conserved positions in the olfactory bulb, needs to be tested for more than a single OR gene. The Nrp1 model (Imai et al., 2006, 2009; Luo, 2015) was developed for one OR (rat OR I7), which was either FLAG- or HA-epitope tagged and expressed from a small transgenic mouse MOR23 promoter, and glomeruli were examined solely for the medial aspect of the bulb. In the intervening decade, the Nrp1 model has not been extended to an endogenous mouse OR, or to any other OR. We have here sought to assess the validity of the Nrp1 model for another population of OSNs, which express mouse OR M71 from the endogenous *M71* locus, using our publicly available, gene-targeted mouse strains. The configurations of M71 glomeruli that we discern in the conditional *Nrp1* knockouts with two distinct *Nrp1* floxed alleles are not consistent with this Nrp1 model. Moreover, we cannot confirm the simple anterior shift in medial projection sites for the population of OSNs expressing rat OR I7 from a transgenic mouse MOR23 promoter; the lateral projection sites, which were not examined (Imai et al., 2006, 2009), typically undergo a ventral shift, and less of an anterior shift.

### The Nrp1 model for anterior-posterior positioning of glomeruli does not apply to the native population of OSNs expressing mouse OR M71 from the endogenous *M71* locus

We observed multiple configurations and substantial variability in the patterns of labeled glomeruli when Nrp1 was knocked out conditionally in M71^+^ OSNs. Further examination may reveal additional configurations or subconfigurations.

We discerned four configurations in triple-mutant n5247MRZ mice, in which all M71^+^ OSNs are Nrp1^–^. In quadruple-mutant NMRCZ mice, in which half of M71^+^ OSNs are Nrp1^+^ and the other half are Nrp1^–^, we discern five configurations, of which three are shared with n5247MRZ mice. A common observation is the occurrence of novel, ectopic glomeruli, anteriorly and/or dorsally. The ectopic anterior glomeruli are formed by ∼20% of Nrp1^–^ M71^+^ OSNs. Their far-anterior ectopic location is strikingly reminiscent of the ectopic anterior glomeruli reported by two groups in constitutive Adcy3 knockout mice, for M71 (Zou et al., 2007) and for M72 (Dal Col et al., 2007), an OR that is highly related to M71. The lack of Nrp1 expression in Adcy3 knockout mice (Dal Col et al., 2007) suggests a possible mechanistic link between OR expression, cAMP generation through Adcy3, and Nrp1 expression.

Our experiments on M71 with two *Nrp1* floxed alleles, including the one used by Imai et al. (2009), are not consistent with the model, in that we did not observe a simple anterior shift of M71 glomeruli when Nrp1 was knocked out conditionally in M71^+^ OSNs. The single large ectopic dorsal glomerulus that we observed frequently was not shifted along an anterior-posterior axis at all, but resided halfway between the medial and lateral M71 glomeruli.

### The Nrp1 model for anterior-posterior positioning of glomeruli does not apply to the novel population of OSNs expressing rat OR I7 from a transgenic mouse MOR23 promoter

During the course of this project, we realized that we needed to take a step back and seek to reproduce the core original findings with which the Nrp1 model was formulated (Imai et al., 2009). With publicly available strains, we generated the same cross of Cre driver strain and *Nrp1* floxed allele, but our observations differed. Instead of a single projection site (glomerulus) shifting anteriorly at PD14, we observed multiple glomeruli formed by the coalescence of axons expressing rat OR I7 from a transgenic mouse MOR23 promoter. When viewed in a merged bulb after 3D construction, together these medial glomeruli occupied a broad domain of the bulb that can best be characterized as a sector that is anchored posteriorly on the tightly clustered medial glomeruli in the transgenic mice with Nrp1. This sector radiates anteriorly and ventrally along various vectors and is not consistent with a simple shift along an anterior-posterior “axis” of the bulb; at best, the vectors have an anterior component. This sector may reflect the domain of the bulb within which these axons, now devoid of Nrp1, are allowed to navigate. We then proceeded to flip these bulbs and examined the labeled glomeruli in the lateral aspect, which were not documented in Imai et al. (2006, 2009). We discovered that the scattering of the glomeruli is even more pronounced in the lateral than in the medial aspect. Together, these lateral glomeruli occupy a domain of the bulb that can be regarded as a belt or a band, centered at the tightly clustered lateral glomeruli in the mice with Nrp1 and extending toward the medial aspect along the ventral ridge. Seven of 24 lateral glomeruli were shifted dorsally compared with the lateral glomeruli in the mice with Nrp1, but most (14/24) were shifted ventrally; the remaining three glomeruli showed no shift. No glomeruli underwent a simple shift along an anterior-posterior “axis” of the bulb.

At PD21, the scattering of glomeruli appears to have increased, with glomeruli radiating further within the sector (medially) and the belt (laterally). We have not examined mice at older ages. There may thus be a phenomenon of dynamic instability when mechanisms of axonal coalescence of OSNs are perturbed by a conditional *Nrp1* knockout in one particular population of OSNs. In this context, it must be kept in mind that the glomeruli that are formed by the coalescence of axons expressing rat OR I7 from a transgenic mouse MOR23 promoter are novel and artificial: they do not exist in wild-type, nontransgenic mice.

Possible explanations for the discrepancy in our findings with Imai et al. (2006, 2009), as far as the medial aspect of the bulb is concerned, are a higher quality of our epifluorescence whole-mount imaging and the new method of 3D reconstruction with serial block-face two-photon tomography (Zapiec and Mombaerts, 2015). Unfortunately, the lateral aspect of the bulb was not examined in Imai et al. (2006, 2009).

### Assessment of Nrp1 gradients in the bulb

It is intrinsically difficult to provide convincing evidence of a Nrp1 protein gradient across the bulb, extending smoothly from anterior-low to posterior-high, at various dorsal-ventral levels, both in the medial and lateral aspects of the bulb, and at relevant ages. We have here tested the concept of a Nrp1 gradient in two ways, and with the same Nrp1 antibody as used in Imai et al. (2006, 2009). By conventional immunofluorescence, we examined three posterior domains at several dorsal-ventral levels in the vicinity of Gucy1b2^+^ glomeruli, which are Adcy3^–^ and Nrp1^–^. We found that there are Nrp1^+^ and Nrp1^–^ glomeruli in these posterior domains, thus breaking the link between Adcy3 and Nrp1 expression, and constituting numerous exceptions to the notion that posterior glomeruli are Nrp1^+^. Moreover, there is substantial heterogeneity within these posterior domains: some glomeruli adjacent to Gucy1b2^+^ glomeruli are Nrp1^+^ (and Adcy3^+^), and others are Nrp1^–^ (but also Adcy3^+^). Serial block-face two-photon tomography (Zapiec and Mombaerts, 2015) is an advanced imaging method, enabling us to quantify Nrp1 immunofluorescence levels in each of the thousands of glomeruli of the bulb. With this method, we were unable to identify consistent, robust, smooth gradients of Nrp1 across glomeruli along any axis, including possible anterior-posterior axes; but the absence of evidence for a bulb-wide gradient in our hands is not evidence for its absence. Our 3D reconstructions reiterate the difficulty in the unambiguous definition of any “axis” in the olfactory bulb (Zapiec and Mombaerts, 2015): there are no landmarks or fiduciary points to determine “anterior” and “posterior” accurately and reproducibly and to the level of precision that is required to assess gradients rigorously and critically. Nonetheless, we found that, generally, anterior glomeruli are Nrp1^low^, and generally, the posterior bulb has glomeruli that are Nrp1^high^. But Nrp1 levels do not form a simple gradient from anterior-low to posterior-high throughout the bulb. Instead the Nrp1 pattern is complex and can better be described as mosaic or patchy, or even idiosyncratic, with the posterior bulb hosting glomeruli that are strongly Nrp1^+^ but intermingled with Nrp1^low^ glomeruli.

### Glomerular Nrp1 levels in the literature

Our observations of mosaic, patchy patterns of Nrp1 levels across glomeruli in sections and in the 3D reconstructed bulbs are consistent with the literature. An early study in rats reported that “[n]europilin-1 immunoreactivity was confined to the rostrolateral and caudal glomeruli of the main olfactory bulb. Moderate neuropilin-1 immunoreactivity was observed in most glomeruli, whereas a small number of glomeruli was either very strongly labeled or unlabeled” (Pasterkamp et al., 1998). Another group reported in adult mice that “NP-1^+^ olfactory axons projected selectively to glomeruli within two compartments, the lateral band … and the medial band.… NP-1^+^ glomeruli (which receive NP-1^+^ olfactory axons) were completely excluded from the anteromedial region … and the ventral region” (Taniguchi et al., 2003). A third group observed in PD5 mice that “Npn-1^+^ glomeruli in the rostral-most 600 µm of the olfactory bulb were variable in size and intensity of staining” and “[a]t no position along the entire rostrocaudal axis of the olfactory bulb were there detectable glomeruli containing npn-1^+^ axons near the ventral midline of the olfactory bulb of wild-type mice” (Schwarting et al., 2004). The Nrp1 patterns in mice were studied in detail in an attempt to explain the phenotype of mice with a knockout of Sema3A, a ligand for Nrp1 (Schwarting et al., 2000, 2004; Taniguchi et al., 2003): Sema3A at the ventral midline would guide Nrp1^+^ axons to regions in the lateral and medial bulb. Dibattista and Reisert (2016) wrote, “we observed a mosaic pattern of Nrp1 expression in sagittal section of the [olfactory bulb]. Glomeruli showed high, low, or even absent levels of Nrp1.” Thus, at least four groups have reported on Nrp1 levels in glomeruli of rat and mouse since 1998, and like ourselves, none have identified a gradient or gradient-like pattern of Nrp1 levels throughout the bulb.

### What, then, may Nrp1 do in axonal wiring of OSNs?

It is puzzling that there are no clear commonalities in the phenotypes of the conditional *Nrp1* knockout between the two populations of OSNs that we studied, although both populations reside in the dorsal main olfactory epithelium and project their axons to glomeruli in the dorsal bulb. When a Cre driver strain for another OR gene becomes available, it is possible that yet another spectrum of phenotypes will be observed. Regardless how strong the effects of the conditional *Nrp1* knockouts are on the number and positions of glomeruli, our observations for these two populations of OSNs cannot be interpreted in terms of Nrp1 levels determining glomerular positions along the anterior-posterior axis. Our findings leave unanswered the broader question as to whether and how OR-specific cAMP signals direct axonal targeting in the olfactory system.

A similar variability for the same *Nrp1* floxed allele (Gu et al., 2003) was reported for axons of chemosensory neurons from the Grueneberg ganglion (a specialized olfactory subsystem) that express *Gucy2g* (Matsuo et al., 2012). One explanation is that in the absence of Nrp1, OSN axons are free to enter into or navigate across otherwise restricted regions of the bulb (such as regions where Sema3A is present), resulting in multiple options for sites where Nrp1^–^ axons can coalesce into glomeruli. We speculate that for OSNs expressing rat OR I7 from a transgenic mouse MOR23 promoter, the sector and belt represent the degree of freedom in the medial and lateral aspects of the bulb that a conditional Nrp1 knockout affords to these axons. In the absence of Nrp1, the positional variability of glomeruli that is observed normally (Zapiec and Mombaerts, 2015) is greatly increased, but without a clear set of rules between medial and lateral aspects, and between the two populations of OSNs that we studied. In any case, Nrp1 contributes to reducing the complexity of the challenging task for axons of OSNs expressing a given OR to coalesce into one or a few glomeruli in highly restricted domains on the bulb.

## Conclusion

Taken together, our results with gene-targeted mice expressing mouse OR M71 from the endogenous *M71* locus and transgenic mice expressing rat OR I7 from a mouse *MOR23* promoter pose a challenge to the Nrp1 model that was formulated by Imai et al. (2006, 2009) and that has made it into a textbook (Luo, 2015). A revision, reformulation, or refinement of this model becomes thus imperative. The lack of public availability of Cre driver strains for other OR genes, in particular gene-targeted strains, precludes additional experiments with conditional *Nrp1* knockouts. Our analyses of Nrp1 levels in 3D reconstructed bulbs and a survey of the literature do not provide evidence for smooth, continuous, or fine-grained gradients of Nrp1 along any axis of the bulb, including along anterior-posterior axes. Moreover, it remains difficult to define and to measure “the” anterior-posterior axis of the bulb in a meaningful and comparable manner. We deem it unlikely that Nrp1 determines positioning in a simple, straightforward, and uniform fashion along any axis for the 3600 glomeruli of the mouse olfactory bulb. Nonetheless we hope that our intriguing observations of a sector and belt of scattered glomeruli in I7-Cre-YFP Tg × n5247-flox mice may provide clues about the mechanisms whereby a conditional *Nrp1* knockout exerts such strong effects on the axonal coalescence of OSNs that express the same OR.

*Note added in proof:* while this paper was under review, Assens et al. (PMID 27578798) reported data that are overlapping and consistent with our data, interpretation, and conclusions.

## Synthesis

The decision was a result of the Reviewing Editor Piali Sengupta and the peer reviewers coming together and discussing their recommendations until a consensus was reached. A fact-based synthesis statement explaining their decision and outlining what is needed to prepare a revision is listed below. The following reviewer(s) agreed to reveal their identity: Diego Restrepo

Both reviewers agree that findings reported in this manuscript will be of general interest to scientists interested in the very intriguing question of the mechanisms by which OSNs expressing specific ORs are targeted to specific glomeruli in the OB. In particular, results in this work potentially refute the model proposed by Sakano's group indicating that glomerular positioning along the A-P axis of the OB is globally mediated by levels of guidance molecules such as Neuropilin-1 regulated by OR-regulated cAMP levels in OSNs.

While all reviewers agree that establishing the alternate mechanism(s) experimentally is beyond the scope of this manuscript, there are several possibilities that can be readily assessed with additional analyses of the current data. In particular, since the conclusions rely on snapshots of the Nrp gradient with associated caveats, these caveats (addressed further below) need to be acknowledged clearly, such that the conclusions are not overstated.

Major:

1. While the results suggest that Nrp1 may not be the sole mediator of axon targeting, do the authors also suggest that cAMP signaling is NOT involved? They need to directly discuss the possibility that while the postulated A-P gradient of Nrp1 may not regulate glomerular positioning, cAMP may still play a role via regulation of additional guidance molecules.

2. Masking glomerular expression of Nrp1 using the VGLUT2 image is recursive. But an argument can be made that for axon targeting the Nrp1 gradient is more relevant in the areas of the olfactory nerve layer overlaying the glomeruli because that is where an important subset of the interactions involved in extending and targeting OSN axons take place. What happens when the Nrp1 signal is masked to show the olfactory nerve area overlaying the glomeruli (VGLUT2 signal)? Is there a more uniform gradient of Nrp1 in the overlaying nerve area?

3. Unfortunately physical section registration problems are evident in the 3D serial block-face two-photon microscopy shown for Nrp1 expression in Figs. 5 and 6. This is particularly evident in Fig. 6B. At the very least the authors should show an image of a single section that clearly demonstrates variability in Nrp1 expression in local areas. In addition, does this lack of registration affect their quantification of Nrp1 levels in Figs 7 and 8? Have the authors quantified expression levels of VGULT2 as a control? The analysis of the serial block 2 photon microscopy is faulty, and would benefit from improvement.

4. The statistical significance of the results shown in Fig. 2 should be tested using pairwise Chi-squared with a correction for multiple comparisons (or another statistical test). In addition, for this figure it is important to show as a control the configuration of glomeruli in M71-IRES-tauGFP mice. Is it possible to also add representative images for the reader to better visualize the glomerular patterns?

5. Could there be multiple roles for Nrp1 at different times (related to the comment regarding snapshots made above)? Thus, could Nrp1 be separately involved in A-P targeting, and in coalescence of smaller ectopic glomeruli? There is abundant literature on multiple ectopic glomeruli for other receptors whose location varies widely through the bulb (particularly for the P2 OR). There is an overall gradient of Nrp1, but as the authors indicate it is highly variable in different neighboring glomeruli. Was the variability in glomerular Nrp1 expression related to the size of the glomeruli? Could Nrp1 be involved in refinement of targeting? Could the mean Nrp1 expression in a space window spanning a few glomeruli be the variable that does affect A-P positioning? Since Nrp1 is knocked out from the M71 glomeruli could there be residual targeting cues from other glomeruli (other axons)?

Minor:

1. Please add genotype labels to each panel in Figure 1 directly. Please also label the glomeruli in Fig 1C.

2. Please add the gene name (Gucy1b2) to GFP so that it is obvious what cells are being marked.

3. What is the relevance of taking the snapshot shown in Fig 6 at P14? How is that related to the time point examined by Imai et al? Is a similar patchy pattern observed at other time points?

4. Please clearly indicate in the Methods and Figure legends the controls that were performed to confirm the specificity of each antibody (or references to previous work if the antibodies have been reported previously).

5. In the manuscript it is stated that: "We find that in n5247MCZ mice at PD22, the EA glomeruli contribute 20.0% (STDEV {plus minus} 6.5%) to the total glomerular volume per mouse. Thus, ∼20% of Nrp1- M71+OSNs innervate these EA glomeruli." State explicitly how the percent of OSNs innervating this glomerulus was estimated and the number of measurements that contribute to this estimate. Also, in Fig. 3 please show an example of olfactory epithelium sections and give details on how the number of OSNs was estimated. Please also provide the results of a statistical test for differences in OSN number in Fig. 3.

6. Fig 5B should be labeled dorsal and not medial.
